# Interpretation of Specific Strength-Over-Resistivity Ratio in Cu Alloys

**DOI:** 10.3390/ma14237150

**Published:** 2021-11-24

**Authors:** Hongming Li, Shuang Zhang, Yajun Zhao, Xiaona Li, Fushi Jiang, Chuang Dong

**Affiliations:** 1 School of Mathematics and Physics, Inner Mongolia Minzu University, Tongliao 028000, China; hongmingli06@126.com (H.L.); jiangfushi@126.com (F.J.); 2 School of Materials Science and Engineering, Dalian Jiaotong University, Dalian 116028, China; yajunzhao@djtu.edu.cn (Y.Z.); dong@dlut.edu.cn (C.D.); 3 Key Laboratory of Materials Modification by Laser, Ion and Electron Beams, Dalian University of Technology, Ministry of Education, Dalian 116024, China; lixiaona@dlut.edu.cn

**Keywords:** Cu alloys, dual-phase mechanical model, strength-over-resistivity ratio

## Abstract

Reaching simultaneously high mechanical strength and low electrical resistivity is difficult as both properties are based on similar microstructural mechanisms. In our previous work, a new parameter, the tensile strength-over-electrical resistivity ratio, is proposed to evaluate the matching of the two properties in Cu alloys. A specific ratio of 310 × 10^8^ MPa·Ω^−1^·m^−1^, independent of the alloy system and thermal history, is obtained from Cu-Ni-Mo alloys, which actually points to the lower limit of prevailing Cu alloys possessing high strength and low resistivity. The present paper explores the origin of this specific ratio by introducing the dual-phase mechanical model of composite materials, assuming that the precipitate particles are mechanically mixed in the Cu solid solution matrix. The strength and resistivity of an alloy are respectively in series and parallel connections to those of the matrix and the precipitate. After ideally matching the contributions from the matrix and the precipitate, the alloy should at least reach half of the resistivity of pure Cu, i.e., 50%IACS, which is the lower limit for industrially accepted highly conductive Cu alloys. Under this condition, the specific 310 ratio is related to the precipitate-over-matrix ratios for strength and resistivity, which are both two times those of pure Cu.

## 1. Introduction

Electrically conductive Cu alloys are generally required to possess sufficient mechanical strength and high conductivity, and the combination of the two is dependent on the material chemistry, which generates a special microstructure after manufacturing [1,2,3,4]. However, due to the contradiction between conductivity and strength [5,6,7], which is complicated by alloying and processing, it is difficult to judge the general performance of an alloy whose conductivity and strength vary inversely. Therefore, the contradictive changes of the two key properties constitute a big challenge for material selection in practice, and a parameter that describes the intrinsic coupling of the two properties is needed for this purpose.

In our previous work, via introducing the cluster-plus-glue-atom model for solid solutions, the coupled variation in electrical conductivity and mechanical strength, issued from chemical alloying, is related to the common structural mechanism of short-range chemical ordering [8,9]. By taking Cu-Ni-Mo alloys as the example, the increments due to alloying (i.e., with respect to pure Cu) in tensile strength (the work hardening in Cu alloys is quite weak, so tensile strength and yield strength are close) and electrical resistivity are linearly correlated with the solute contents [10,11]. Therefore, a new parameter is generated after eliminating the solute contents in both expressions, which is the ratio of the tensile strength increase relative to pure Cu over the residual electrical resistivity, hereafter called the strength/resistivity ratio. This ratio reflects the comprehensive strength and resistivity performance, whereby a large ratio indicates a highly conductive alloy with superior mechanical strength. On the other hand, each Cu alloy system falls within a narrow ratio range. In particular, we have pointed out that a specific ratio of 310 × 10^8^ MPa·Ω^−1^·m^−1^ distinguishes commonly accepted high-strength and conductive Cu alloys from those normally regarded as structural ones [12], indicating the system-independent helpful characteristic used to evaluate the alloy performance quantitatively.

In the following, our previous work on the strength/resistivity ratio is first briefly presented, focusing on the cluster-plus-glue-atom model from which the relations between the strength and resistivity properties and the alloy compositions are established. Then, the dual-phase mechanical model of composite materials is used to explore the physical origin of the specific ratio of 310 × 10^8^ MPa·Ω^−1^·m^−1^, hereafter called the 310 ratio for simplicity.

## 2. Definition of Strength/Resistivity Ratio

### 2.1. Cluster-Plus-Glue-Atom Model to Describe the Short-Range Chemical Ordering in Cu-Ni-Mo Alloys

Under similar processing conditions (i.e., the structural defects are nearly the same), the strength and resistivity of a Cu alloy depend on the microstructure state, characterized by precipitation in an almost purified Cu matrix. The typical heat treatment involves a solution in a single-phase state plus ageing for precipitation. Therefore, the structural evolution is always traced to the parent single-phase solid solution state.

Solid solutions are characterized by heterogeneous local structure formation, called short-range chemical ordering. As the embryos of precipitation, this special local ordering is also mixed with certain disordering, which makes its modeling difficult. Electrical resistivity and mechanical strength increments due to alloying are related to this [12]. As with precipitates, short-range chemical orders scatter conducting electrons and at the same time constitute obstacles to dislocation movement. For this reason, strength and resistivity coupling is always present in solid solutions, even after the precipitation occurs. The key to improving the performance is the proper control of the short-range chemical ordering state and henceforth the precipitation. A structural model is, therefore, necessary to quantify the local orders.

This is made possible by introducing our cluster-plus-glue-atom model for solid solutions [13,14]. Short-range ordering is formed due to the charge shielding around any given atom that results in oscillating distribution of the electron density, namely Friedel oscillations [15]. This oscillating behavior of the electrons in turn causes the same oscillation of the atomic density, which is prominent over short ranges, especially in the nearest and next-nearest neighborhoods. Local units, showing charge neutrality and mean density, can be defined using certain cut-off distances, the smallest of which covering only the nearest-neighbor *cluster* and a few next-neighbor *glue atoms* [14]. This model simplifies any structure into a local unit, expressed in cluster formula form as [*cluster*] (*glue atoms*) [13]. We have shown by analyzing many industrial alloys that popular alloys are all based on simple cluster-plus-glue-atom formulas, such as [Zn-Cu_12_]Zn_4_ for brass Cu-30Zn, [Ni-Fe_12_]Cr_2_(Ni,Nb,Ti)_1_ for maraging stainless steel Custom465, etc. [14].

**Figure 1:**
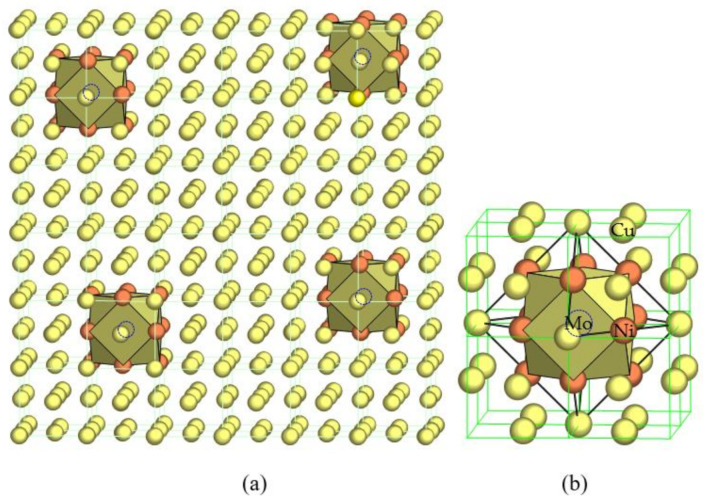
Cluster-plus-glue-atom model for a stable solid solution in an FCC–Cu alloy, where immiscible solute Mo (hidden in the cuboctahedra, marked by open circles) is made in solution with Cu via the intermediate of miscible solute Ni (solid orange circles) by forming Mo-centered and Ni-nearest-neighbored clusters scattered in Cu (solid yellow circles) matrix (**a**) and locally enlarged (**b**), as initially proposed in [10].

Let us take the modeling of the short-range chemical ordering in face-centered cubic Cu-Ni-Mo solid solutions as an example. Considering the relatively strong and negative interactions between Mo and Ni, a Mo-centered and Ni-shelled cuboctahedral cluster should show the ideal stable local structure, [Mo_1_-Ni_12_], which will be scattered in the Cu solid solution matrix, as shown in Figure 1 [10]. Mo, which is immiscible with Cu, will now be contained in complete solution via an intermediate of Ni that is miscible with both Cu and Mo. The structure of the Cu-Ni-Mo solid solution is, therefore, described by a structural unit composed of the cluster plus some Cu atoms as the glue atoms, or expressed as [Mo_1_-Ni_12_]Cu*_x_*. Of course the real local structure is always less ordered and mixed occupancies in the nearest neighbors and in the glue sites should occur, especially at high temperatures. The assumed purely Mo-Ni neighborhood can only be taken as the ideal case when atomic interaction modes are fully satisfied. The formation of such highly ordered clusters more effectively inhibits dislocation movement than less ordered states, and in a similar manner decreases the scattering probability by grouping individual atomic scatters into large clusters. In brief, local ordering, as with precipitation, favors high strength and low resistivity.

### 2.2. Proposition of Strength/Resistivity Ratio

According to the above cluster model, a typical Cu-Ni-Mo alloy is simplified into [Mo_1_-Ni_12_] clusters scattered in a Cu matrix. Any deviation from the ideal cluster formula [Mo_1_-Ni_12_]Cu*_x_* would induce either a solution in Cu of extra Ni or Mo precipitation of extra Mo. Therefore, there would be three ideal microstructural states, namely the [Mo_1_-Ni_12_] cluster solution in a pure Cu matrix for Mo/Ni = 1/12, a cluster solution plus extra Ni solution in Cu for Mo/Ni < 1/12, and a cluster solution plus extra Mo precipitation for Mo/Ni > 1/12. Measurements of the microhardness and electrical resistivity of alloy series with various Mo/Ni ratios and total solute contents have been conducted, as reported in [11]. The measured electrical resistivity and microhardness data are correlated with these three structural states to reveal the property dependencies on solute contents. Analogous to residual resistivity, which indicates the change of resistivity with reference to pure Cu, the residual microhardness can also be defined. For the ideal cluster solution state (Mo/Ni = 1/12), the residual resistivity is related to the residual microhardness by a factor of 0.72. Such simple relationships indicate that resistivity and strength increments due to solute additions are dependent on the same cluster–solution mechanism and can be a good reference for evaluating the strength and resistivity performance of Cu alloys.

To simplify the description of the strength and resistivity coupling, we introduced a new parameter, the so-called strength/resistivity ratio [12], referring to the ratio of strength and resistivity increments purely due to alloying (i.e., concerning pure Cu, ignoring structural defects due to deformation processing). Microhardness values are abundantly available and are converted into tensile strength values following [6].

In the ideal case where Mo precipitation occurs in a pure Cu matrix, the optimum combination of strength and resistivity would be reached. Since the strength and resistivity increments are proportional to the amount of Mo precipitation by factors of 28 MPa (= 8.4 HV × 10/3) and 0.09 × 10^−8^ Ω·m, respectively, the strength/resistivity ratio in correspondence to this ideal structural state is about 310 × 10^8^ MPa·Ω^−1^·m^−1^, which is the highest possible ratio for Cu-Ni-Mo alloys. It should be mentioned that regardless of how the dislocations are blocked, following particle shearing or Orowan dislocation looping, macroscopically the strength will be proportional to the density of chemical short-range-order clusters and precipitates. In a similar manner, conduction electrons are scattered. Therefore, the residual strength and residual resistivity are somehow linked, from which the specific strength/resistivity ratio arises.

In the resistivity–strength plot [12], it can be noted that this specific ratio of 310 × 10^8^ MPa·Ω^−1^·m^−1^ actually points to the boundary separating high-strength and conductive Cu alloys from structural Cu alloys. This means that although this specific 310 ratio is derived from Cu-Ni-Mo alloys prepared under identical conditions [12], it is in fact system independent and should indicate a certain structural mechanism. Next, we will illustrate how to understand this ratio by introducing the dual-phase mechanical model of composite materials.

## 3. Dual-Phase Mechanical Model for the Specific Strength/Resistivity Ratio

### 3.1. Dual-Phase Mechanical Model of Composite Materials

We are confined to addressing a composite system containing two phases, the matrix and strengthening particles. Ignoring interface effects (mechanical mixing) and regarding the composite as homogeneous, the rule of mixtures [16], mimicking Végard’s law [17], generally applies when predicting strength, modulus, resistivity, others: the composite property is dependent linearly on the volume fraction and the property of the dual phases.

The tensile strength σ of the composite is then expressed by those of the dual phases and their volume fraction *f* ’s [18] as:

*σ* = *σ*_0_ · (1 ‒ *f*_p_) + *σ*_p_ · *f*_p_ (1)

where the subscripts 0 and p refer respectively to pure Cu and precipitate.

According to Karasek and Verhoeven [19,20], the electrical resistivity *ρ* of the composite satisfies a parallel relationship with those of the dual phases:

1/*ρ* = (1 ‒ *f*_p_)/*ρ*_0_ + *f*_P_/*ρ*_P_ (2)

### 3.2. Ideal Strength Matching to Reach 50%IACS

Multiplying Equation (1) by (2) generates *σ*/*ρ* = *σ*_0_ · (1 – *f*_p_)^2^/*ρ*_0_ + *σ*_p_ · (*f*_p_ – *f*_p_^2^)/*ρ*_0_ + *σ*_0_ · (*f*_p_ – *f*_p_^2^)/*ρ*_P_ + *σ*_p_ · *f*_p_^2^/*ρ*_P_. Since the precipitate fraction *f*_p_ is always a minor quantity, the *f*_p_^2^ term can be ignored and the above product is simplified into:

*σ*/*ρ* = *σ*_0_ · (1 − 2*f*_p_)/*ρ*_0_ + *σ*_p_ · *f*_p_/*ρ*_0_ + *σ*_0_ · *f*_p_/*ρ*_p_ = *σ*_0_/*ρ*_0_ + *σ*_0_ · *f*_p_/*ρ*_p_ + *f*_p_ · (*σ*_p_ − 2*σ*_0_)/*ρ*_0_ (3)

To maximize *σ*/*ρ*, the contributions of the three terms should all be positive, which requires *σ*_p_ ≥ 2*σ*_0_, i.e., the strength of the precipitate phase *σ*_p_ must be at least twice that of pure Cu. Let *σ*_p_ = (*n* + 1)*σ*_0_, then *n* ≥ 1 is the index to show the strength level of the precipitate relative to pure Cu, where *n* = 1 (*σ*_p_ = 2*σ*_0_) constitutes the limiting condition for high-strength and conductive alloys. We then examine the expression of the strength/resistivity ratio by taking *σ*_p_ = 2*σ*_0_ as the prerequisite.

### 3.3. Strength/Resistivity Ratio

The strength/resistivity ratio, defined as (*σ* − *σ*_0_)/(*ρ* − *ρ*_0_), reflects the increment of strength relative to resistivity concerning pure Cu.

The electrical conductivity of Cu alloys is commonly expressed compared to that of standard pure Cu at *ρ*_0_ = 1.75 × 10^−8^ Ω·m, or IACS = *ρ*_0_/*ρ* (International Annealed Copper Standard for conductivity). Further combining Equation (1) into (*σ* – *σ*_0_)/(*ρ* – *ρ*_0_) leads to:

(4) $$\frac{\sigma -\sigma_{0}}{\rho -\rho_{0}}=\frac{\sigma_{P}-\sigma_{0}}{\rho -\rho_{0}}\cdot f_{P}=\frac{n\cdot f_{P}}{\frac{1}{\mathrm{IACS}}-1}\cdot \frac{\sigma_{0}}{\rho_{0}}$$

Following Equation (4), in order to make the ratio larger than that of pure Cu, then $n\cdot f_{P}\geq \frac{1}{\mathrm{IACS}}-1$ or $\mathrm{IACS}\geq \frac{1}{n\cdot f_{P}+1}$. Since *f*_p_ ≤ 1, $\mathrm{IACS}\geq \frac{1}{n\cdot f_{P}+1}\geq \frac{1}{n+1}$. When *n* = 1, i.e., *σ*_p_ = 2*σ*_0_, IACS ≥ 0.5. This last deduction means that when *σ*_p_ = 2*σ*_0_, the conductivity should be at least 50%IACS for alloys featuring high strength and conductivity.

Under the condition of IACS ≥ 0.5, *n*·*f*_P_ ≥ 1 provides the criterion to judge the volume fraction of the precipitates. For example, in the Cu-Mo binary system, the precipitate is elemental Mo, with a tensile strength of 580 MPa, so that *σ*_p_ ≈ 2.15*σ*_0_ and *n* = 1.15. Then, *f*_P_ ≥ 0.87, which indicates an overwhelming presence of Mo precipitation. Therefore, Cu-rich Cu-Mo alloys cannot simultaneously reach high strength and conductivity. For Cu-Cr alloys, the precipitate is elemental Cr with a microhardness of 1300 HV [21], so that *σ*_p_ ≈ 15.99*σ*_0_ and *n* = 14.99. Then, *n*·*f*_P_ ≥ 1 gives *f*_P_ ≥ 0.067, indicating that a minor amount of Cr precipitation would reach high strength and conductivity. In fact, Cu-Cr alloys are indeed well-known for their high strength in combination with their high conductivity.

The above discussion focuses on the strength part. Next, we will examine the resistivity part. Assuming the resistivity of pure Cu *ρ*_0_ to be *k* times the resistivity of the precipitate *ρ*_p_, i.e., *ρ*_0_ = *k*·*ρ*_P_ (0 < *k* ≤ 1, reflecting the electrical resistivity of pure Cu relative to that of the precipitate phase), Equation (4) becomes $\frac{\sigma -\sigma_{0}}{\rho -\rho_{0}}=\frac{\sigma_{0}}{\rho_{0}}\cdot (\frac{n}{1-k}-n\cdot f_{P})$. For Cu alloys with high strength and conductivity, *n* ≥ 1 and *f*_P_ is a minor quantity, while the strength/resistivity ratio is expressed as:

(5) $$\frac{\sigma -\sigma_{0}}{\rho -\rho_{0}}=\frac{\sigma_{0}}{\rho_{0}}(\frac{n}{1-k}-n\cdot f_{P})\geq \frac{\sigma_{0}}{\rho_{0}}(\frac{1}{1-k}-f_{P})>\frac{\sigma_{0}}{\rho_{0}}\cdot \frac{1}{1-k}$$

According to Equation (5), when *f*_P_ is a minor quantity, the ratio is approximately expressed as the strength/resistivity ratio of pure Cu by divided (1 − *k*). Since *k* = *ρ*_0_/*ρ*_P_ < *ρ*_0_/*ρ* = IACS, *k* is in the range of 0~IACS. As far as the conductivity is concerned, a larger *k* increases the ratio more effectively. When *k* is larger than the lower limit for the Cu alloy featuring high strength and conductivity, IACS = 0.5, or *k* > 0.5, the resistivity of precipitate is more than twice of that of pure Cu, *ρ*_P_ > 2*ρ*_0_. Equation (5) then becomes:

(6) $$\frac{\sigma -\sigma_{0}}{\rho -\rho_{0}}>2\frac{\sigma_{0}}{\rho_{0}}$$

This equation states that under the condition of *σ*_p_ = 2*σ*_0_ and *ρ*_p_ = 2*ρ*_0_, the lower limit of the strength/resistivity ratio is twice that of pure Cu. This critical ratio, derived from the simple mechanical mixing of dual phases, is actually alloy-independent and is related only to that of pure Cu by a factor of 2.

For pure Cu, the room-temperature resistivity is *ρ*_0_ = 1.75 × 10^−8^ Ω·m, while the tensile strength of the soft state (without cold working) is 230~290 MPa [22]. Its strength/resistivity ratio *σ*_0_/*ρ*_0_ falls in 126~166 × 10^8^ MPa·Ω^−1^·m^−1^. Twice this value gives the lower limit for Cu alloys having high strength and low resistivity of 263~331 × 10^8^ MPa·Ω^-1^·m^-1^. The specific 310 ratio as deduced from the Cu-Ni-Mo alloy is in this range. 

Cu-Ni-Mo alloys are composed of a Cu solid solution matrix plus elemental Mo precipitation in a non-coherent manner, meaning the dual-phase mechanical mixing should apply. At room temperature, the tensile strength of Mo is 580 MPa, which is almost twice that of pure Cu in the soft state, or *σ*_p_ = 2*σ*_0_, indicating that this alloy system could possibly be a candidate for reaching high strength and low resistivity. However, the resistivity of Mo is 5.6 × 10^−8^ Ω·m, nearly three times that of pure Cu, or *ρ*_p_ = 3*ρ*_0_, meaning *k* ≈ 0.3 < 0.5, falling beyond the requirement for high conductivity.

In the above discussions, we ignore the influences from structural defects such as grain boundaries, twinning, dislocation, point defects, etc. All of these defects would increase the strength/resistivity ratio. Therefore, the deduced 310 ratio can only be the lower limit for Cu alloys featuring high strength and low resistivity.

## 4. Conclusions

The present work utilized the basic model of composite materials, the dual-phase mechanical mixing, to unveil the microstructural mechanism for the critical strength/resistivity ratio of 310 × 10^8^ MPa·Ω^−1^·m^−1^ that constitutes the lower limit of Cu alloys with high strength and conductivity. The strength and resistivity of a dual-phase system are dependent linearly on the volume fractions and the properties of the matrix and the precipitate phases. After ideally matching the contributions from the matrix and the precipitate, the alloy should at least reach half of the resistivity of pure Cu, i.e., 50%IACS, which is the lower limit for industrially accepted highly conductive Cu alloys. Under this condition, the specific ratio is related to the precipitate/matrix ratios for strength and resistivity, which are both two times those of pure Cu.

## Data Availability

The data underlying this article will be shared on reasonable request from the corresponding author.
